# Beef Tallow‐Based Skincare Claims in Social Media: A Cross‐Sectional Analysis

**DOI:** 10.1111/jocd.70544

**Published:** 2025-11-28

**Authors:** Lanah Almatroud, Sooin Choi, Karissa Libson, Kurt Ashack

**Affiliations:** ^1^ College of Human Medicine Michigan State University East Lansing Michigan USA; ^2^ Transitional Year Residency Corewell Health/Michigan State University Grand Rapids Michigan USA; ^3^ Dermatology Associates of West Michigan Kentwood Michigan USA

**Keywords:** animal‐based skincare, beef tallow, tallow skincare

## Abstract

**Background:**

Beef tallow, or “tallow”, is a solid fat derived from animals with a high content of triglycerides and essential fatty acids. Tallow has gained popularity as a skincare product on social media in recent years, driven by the increasing demand for natural and sustainable beauty solutions. There are few studies investigating tallow as a skincare ingredient and its effects on dermatological conditions.

**Aims:**

To promote responsible use of tallow by characterizing information about tallow on social media platforms, examining the quality of claims made in the context of available evidence, and evaluating areas of financial bias.

**Methods:**

Social media posts from various platforms (YouTube, Instagram, and TikTok) were evaluated in a cross‐sectional analysis to assess claims about tallow‐related skin care, the quality of information provided, and potential financial biases.

**Results:**

Claims of efficacy for acne, atopic dermatitis, and psoriasis were prevalent but largely lacked cited evidence. The majority of the posts promoting beef tallow across platforms demonstrated financial bias and were uploaded by individuals lacking credentials in healthcare. Additionally, most existing evidence for tallow use is based on studies examining its ingredients.

**Conclusion:**

Despite growing anecdotal claims that beef tallow benefits skincare and dermatologic conditions, evidence remains insufficient to support these claims. Many promotions of beef tallow for skin care are associated with financial bias. Further research is needed to evaluate its long‐term effects, ideal formulations, and suitability for different skin conditions.

## 1 Introduction

Tallow, a solid fat derived from animals, is increasingly being used in skincare products due to its high content of triglycerides, essential fatty acids, and fat‐soluble vitamins which benefit the skin barrier. This research focuses on tallow isolated from cows, or “beef tallow”.

Both tallow and human sebum have high proportions of palmitic acid and oleic acid, allowing tallow to integrate into the skin's lipid matrix to support barrier function [1, 2]. Fatty acids and triglycerides act as vehicles to enhance the uptake of other beneficial compounds into keratinocytes, improving skin health [3, 4]. Additionally, natural oils and fats, including those derived from tallow, have been shown to enhance skin penetration and hydration, making them suitable for use in cosmetic emulsions [5, 6]. The use of natural lipids in skincare formulations is supported by their biocompatibility and ability to improve skin barrier function [7]. Moreover, the presence of fat‐soluble vitamins in tallow further amplifies its potential benefits, enhancing its ability to support skin health at a cellular level.

Tallow‐based products have gained significant popularity in both online commercials and homemade skin care communities, driven by the increasing demand for natural and sustainable beauty solutions. While the scoping review by Russell et al. [8] underscores the need for further research to validate the clinical efficacy of tallow in skincare formulations, anecdotal evidence and personal testimonials on social media platforms have fueled its rise. Social media has become a primary source of information for individuals seeking guidance on skincare treatments, products, and conditions.

In this paper, we examine the current landscape of beef tallow in social media posts across popular platforms such as YouTube, Instagram, and TikTok. We characterize claims made, examine the quality of information provided, identify the presence of financial bias, and explore the alignment or lack thereof between these promotional assertions and evidence‐based knowledge. To evaluate how these anecdotal claims are represented in public discourse, we analyzed social media content across multiple platforms.

## 2 Methods

On December 19, 2024, we conducted a cross‐sectional analysis by compiling a list of the first 50 YouTube (YT) videos and 50 YouTube shorts (YS) resulting from searching “tallow skin.” This search was conducted in an incognito browser window to minimize tailored suggestions, and videos were sorted by relevance. The videos were watched and analyzed by two independent reviewers.

On December 31, 2024, an Instagram (IG) search was performed using the hashtag “#tallowskincare” and the top 50 IG video and photograph posts using this hashtag were identified. On the same day, a TikTok (TT) search was conducted using the term “#tallowskincare” filtered by the top 50 TT posts. To minimize tailored content suggestions, new IG and TT accounts were created specifically for this analysis. The IG posts and TT videos were then watched and analyzed by two separate reviewers over the following month.

For all platforms, content in English and with a primary focus on beef tallow for skincare was included. Exclusion criteria included posts or videos in languages other than English, or those focusing on unrelated uses of beef tallow (e.g., cooking). Duplicate posts or videos across platforms were excluded to ensure unique data points.

For each post or video, we collected information to assess viewership and popularity of the post, financial bias, quality of information, and specific claims made. Financial bias was defined as promoting a product with clear indications of a commercial or financial relationship. Indicators of financial bias included the presence of affiliate links, partner discount codes, explicit sponsorship statements, or disclosures of ownership or employment affiliations with specific brands featured in the content. To compare groups based on the presence of financial bias, we conducted a two‐sample *t*‐test assuming unequal variances with two‐sided *p*‐values.

In addition to our primary analysis of social media content, we performed an exploratory narrative search of PubMed for clinical studies, systematic reviews, and meta‐analyses, using the terms “tallow” AND (“skin” OR “skincare” OR “dermatology”). All English‐language articles discussing tallow in relation to skin or dermatologic applications were included. Articles unrelated to skincare or published in non‐English languages were excluded. No date restrictions were applied.

## 3 Results

The data for YouTube Shorts, YouTube videos, Instagram posts, and TikTok videos were characterized across various metrics (Table 1). Risks associated with tallow use were rarely mentioned, with YouTube Videos having the highest rate of risk disclosure (24%). Scientific evidence supporting the claims was minimal, with YouTube Videos being the only platform to cite sources (16%). Furthermore, very few posts recommended consulting a healthcare professional before using tallow (Table 1).

**TABLE 1:** Characteristics of social media posts promoting beef tallow for skincare across YouTube shorts, YouTube videos, Instagram, and TikTok.

	YouTube shorts (*N* = 50)	YouTube videos (*N* = 50)	Instagram posts (*N* = 50)	Tik Tok (*N* = 50)
Sponsored posts	18	15	4	
Median age of post at time of data collection (days)	436 (IQR = 231–577)	342 (IQR 121–864)	10 (IQR = 3–17)	26 (IQR = 10–91)
Median number of views	9576 (IQR = 1708–61 362)	11 667 (IQR = 3261–54 400)	—	181 499 (IQR = 45 125–862 800)
Median number of likes	349 (IQR = 98–1500)	217 (IQR = 41–1825)	10 (IQR = 3–24)	5768 (IQR = 1361–27 500)
Median number of comments	10 (IQR = 3–104)	43 (IQR = 5–179)	1 (IQR = 0–3)	160 (IQR = 47–419)
Median number of saves	—	—	—	1269 (IQR = 222–5901)
Upload source				
Personal channel	34	34	7	39
Beauty or skin care brand	11	14	42	9
Educational organization	1	0	0	1
Other	4	2	1	1
Speaker credentials				
Dermatologist	5	3	0	7
Non‐dermatologist physician (MD/DO)	5	2	0	2
Other	40	45	50	41
Recommended tallow for skincare				
Yes	38	42	45	34
No	9	2	0	11
Neutral	3	6	10	5
Recommended specific product or brand	33	32	45	28
Mentioned risks of use	9	12	0	10
Recommended consulting with healthcare professional before use	0	2	1	1
Cited evidence	1	8	0	0
Demonstrated financial bias	28	37	46	28

82% of posts across all platforms recommended tallow for skincare, with non‐dermatologist physicians and non‐medical speakers endorsing it the most (55% and 83%, respectively). TikTok videos had the highest median views and engagement, while Instagram posts had the least interaction. Sponsored posts were most common on YouTube shorts (36%) and YouTube videos (30%), with Instagram showing the fewest (8%) (Table 1).

Financial bias was prevalent on all platforms, with Instagram (92%) and YouTube videos (74%) showing the highest rates. Tallow was most commonly recommended on Instagram (90%) and YouTube videos (84%), with posts on these platforms also being more likely to mention specific products or brands (Table 1). Many posts endorsing tallow for skincare made claims about its efficacy for conditions like acne (29 posts), eczema (25 posts), and psoriasis (12 posts, Table 2).

**TABLE 2:** Dermatologic conditions and skincare benefits claimed in beef tallow–related social media posts.

Dermatologic condition	Number of videos endorsing benefit (*N* = 200)
*Acne*	29
*Eczema*	25
*Psoriasis*	12
*Rosacea*	1
Moisturizing/protective	26
Anti‐inflammatory	2

Posts with financial bias were significantly more likely to recommend tallow for skincare (82%, *p* < 0.0001), and beauty or skincare brands demonstrated the highest financial bias (96%, *p* < 0.0001). Dermatologists, by contrast, were the least likely to promote tallow (7%) or exhibit financial bias (7%). Among non‐dermatologist physicians and other speakers (such as estheticians and health coaches), financial bias was much more common (67% and 76%, respectively) (*p* = 0.006) (Table 3).

**TABLE 3:** Odds ratios for factors associated with recommending tallow skincare, evidence citation, and financial bias (reference = other [ex: PhD]).

Variable	Recommending tallow skincare, OR (95% CI)	*p*	Evidence cited, OR (95% CI)	*p*	Financially biased, OR (95% CI)	*p*
Dermatologist	0.01 (0.00–0.05)	< 0.001	0.00 (0.00–NaN)^a^	0.71	0.00 (0.00–NaN)^a^	< 0.001
Non‐derm MD/DO	0.07 (0.02–0.30)	0.002	8.63 (1.42–52.54)	0.05	0.56 (0.13–2.37)	0.45
Other (ex: PhD)	1.00 (reference)	NA	1.00 (reference)	NA	1.00 (reference)	NA

^a^ NaN = not a number (upper confidence interval bound not estimable due to model boundary issues).

Across all platforms, most posts were from individuals or bloggers without healthcare or medical expertise, and over half demonstrated financial bias, with beauty and skincare brands showing the highest financial bias (96%) compared to personal channels (55%). Dermatologists demonstrated lower rates of financial bias (7%), while non‐dermatologist physicians and other speakers showed significantly higher rates (67% and 76%, respectively, Table 3).

## 4 Discussion

This study reveals that most social media posts across social media platforms recommended beef tallow for skin care. Further, users cite the benefits of beef tallow's moisturizing, protective, and anti‐inflammatory properties and improvement in skin conditions such as acne, eczema, and psoriasis.

High‐level evidence to support these claims is not available; however, research on fatty acid components of tallow, such as oleic, stearic, linoleic, and palmitic acids, supports its role in barrier recovery of the stratum corneum [2, 4, 9, 10]. Oleic acid (OA), a monounsaturated fatty acid, is a recognized skin penetration enhancer. In vitro ultrastructural work shows that OA disrupts stratum corneum lipid packaging and may increase permeability [6, 11]. Akinshina et al. conversely show that OA may improve hydration [12]. Microbial assays indicate OA inhibits *Cutibacterium acnes* lipase, suggesting potential anti‐acne benefits [13]. Stearic acid supports hydration and skin barrier function without promoting abnormal keratinization in both bilayer modeling and keratinocyte studies [12, 14].

Linoleic acid (LA), although a minor tallow component, shows more consistent anti‐acne and anti‐inflammatory activity: in a clinical study, topical LA reduced microcomedone size, while in vitro sebocyte experiments confirmed its ability to downregulate inflammatory cytokines relative to palmitic acid [15, 16].

However, these lipid components may also worsen acne. Oleic acid may have pro‐inflammatory effects and trigger sebaceous gland hyperactivity, potentially worsening acne [17, 18]. Palmitic acid, a major component of sebum, has been shown to increase pro‐inflammatory cytokines IL‐6 and IL‐8 in sebocytes and can contribute to the pathogenesis of acne by promoting clogged pores [19, 20]. Stearic acid can also provoke inflammation in excess, making it less suitable for oily acne‐prone skin [21]. Given the lipid composition of tallow, its classification as “non‐comedogenic” on social media warrants further studies.

Evidence for tallow benefit in eczema and psoriasis is similarly unclear. In the context of psoriasis, a study by El Mahi et al. [22] found that areas of skin previously affected by psoriasis with higher oleic acid levels had a lower IL‐17‐driven inflammatory response. Additionally, linoleic acid has been demonstrated to decrease pro‐inflammatory cytokines in human monocytic cells and promote wound healing [16, 23, 24].

In atopic dermatitis (AD), restoring lipid profiles is beneficial in principle, but outcomes depend on ex vivo ratios/vehicles [2, 3, 25]. Eczema, which often improves with lipid (ceramide) supplementation, may benefit from formulations containing palmitic and stearic acids which have been shown in an ex vivo tape‐stripped skin model to improve stratum corneum repair by boosting lipid production and transport, thereby strengthening the skin barrier [3]. However, studies in both human and reconstructed‐skin models demonstrate that oleic acid and other FFAs can increase transepidermal water loss over time, which may exacerbate eczema [26, 27].

While rarely mentioned on social media posts in our study, the use of tallow may carry safety risks. For example, beef tallow may not be shelf‐stable and requires proper storage to maintain quality. Animal‐derived fats may be susceptible to microbial contamination during rendering and storage and its high proportion of unsaturated fatty acids increases the risk of rancidity. Further, in patients with atopic dermatitis or sensitive skin, bovine products may induce sensitizing allergenic reactions. Finally, since beef tallow in skincare is used as a cosmetic ingredient, the Food and Drug Administration (FDA) does not require pre‐market approval, so there will be variability in product quality. This research demonstrated 82 unique tallow retailers, each of which may all follow different quality guidelines, if any. These risks highlight the importance of patient counseling and the need for dermatological evaluation of tallow‐based skincare products before giving clinical recommendations.

Additionally, while some components of tallow have potential benefits for skin hydration and inflammation modulation, others raise concerns about acne exacerbation and barrier disruption. The conflicting properties of its fatty acids suggest that tallow may not be universally beneficial and that its effects likely vary based on individual skin type and condition. There is currently insufficient scientific evidence to definitively conclude whether beef tallow is helpful or harmful for acne, psoriasis, and atopic dermatitis and further research is needed.

For individuals who do not have access to dermatological care and those who seek advice through the online community, social media can be an educational tool for dermatologists to spread awareness and direct patients to reputable sources of information [28, 29, 30, 31]. Around half of the posts mentioning risks of tallow use were from dermatologists. In clinical practice, dermatologists can offer evidence‐based guidance on skincare while acknowledging that some anecdotal experiences shared on social media may have merit [32]. This approach can help bridge the gap between evidence‐based practices and personal experiences, encouraging open discussions with patients about their choices.

Existing evidence supporting the use of tallow is heavily based on research studying the fatty acid components of tallow. Future research evaluating the use of tallow itself on skin care and dermatology conditions such as eczema, psoriasis, and acne is needed. Additionally, an established dedicated regulatory body and a standardized set of guidelines for product testing, ingredient safety, and marketing transparency would help protect consumers from misinformation and promote the use of evidence‐based skincare practices.

Limitations of this research include an incomprehensive review of social media posts due to the evaluation of narrow search terms to filter out irrelevant posts and personalized social media algorithms. Additionally, results are limited to the definition of financial bias used in this research which may not completely describe financial/personal incentives in promoting products on social media. Furthermore, the discussion of risks and benefits of tallow for skincare is limited to research on the components of beef tallow. Thus, the findings in this paper should be interpreted as complementary to existing clinical evidence rather than a replacement for it.

## 5 Clinical Perspective and Patient Counseling Scenarios

To bridge research findings with clinical practice, we propose several hypothetical scenarios illustrating how dermatologists might address patient inquiries about beef tallow use in skincare:

1. Acne scenario
  A 20‐year‐old patient presents with moderate acne and asks whether beef tallow, which they saw recommended on TikTok, is a safe alternative to standard treatments.
  - *Clinical approach*: The dermatologist acknowledges the patient's interest, explains that while certain fatty acids in tallow (such as linoleic acid) may have anti‐inflammatory benefits, others, such as oleic and palmitic acid, could exacerbate acne by promoting inflammation and clogged pores. The dermatologist recommends established first‐line acne therapies and discusses potential risks of using unregulated tallow products, while maintaining openness to ongoing research.
2. Eczema scenario
  A parent of a 6‐year‐old with atopic dermatitis reports seeing multiple Instagram posts promoting tallow for eczema relief.
  - *Clinical approach*: The dermatologist explains that while some fatty acids (palmitic and stearic acid) may support skin barrier repair, other components (oleic acid) may worsen barrier function and increase water loss. The dermatologist recommends proven treatments, such as emollients with ceramides, topical corticosteroids when appropriate, cautions about variability and contamination risks in unregulated tallow products, and stresses the importance of allergen sensitivity testing in children with atopic dermatitis.
3. Psoriasis scenario
  A 40‐year‐old patient with stable plaque psoriasis inquires about using a homemade tallow balm after reading anecdotal success stories online.
  - *Clinical approach*: The dermatologist validates the patient's curiosity, notes that evidence supporting fatty acid components (such as linoleic acid reducing pro‐inflammatory cytokines) is preliminary and emphasizes the lack of clinical trials evaluating tallow itself. The dermatologist advises against replacing prescribed treatments with tallow, but if the patient wishes to use it as a complementary moisturizer, stresses the need for careful monitoring of skin reactions and continued adherence to evidence‐based therapies.

## 6 Conclusion

Despite growing anecdotal claims, evidence supporting beef tallow's benefits for skincare and dermatologic conditions remains insufficient. Many promotions of beef tallow for skin care are associated with financial bias. Further research is needed to evaluate its long‐term effects, ideal formulations, and suitability for different skin conditions.

## Author Contributions

Study conception and design was by Dr. Karissa Libson. Literature search, article screening/review, and data extraction were performed by Lanah Almatroud and Sooin Choi. Data analysis was performed by Lanah Almatroud, Sooin Choi, and Dr. Karissa Libson. The final draft of the manuscript was written by Lanah Almatroud, Sooin Choi, Dr. Karissa Libson, and Dr. Kurt Ashack. The final manuscript was reviewed and approved by all authors.

## Conflicts of Interest

The authors declare no conflicts of interest.

## Data Availability

The data that support the findings of this study are available from the corresponding author upon reasonable request.
